# Asthma Control Test and Bronchial Challenge with Exercise in Pediatric Asthma

**DOI:** 10.3389/fped.2016.00016

**Published:** 2016-03-08

**Authors:** Salvatore Tripodi, Mario Barreto, Andrea Di Rienzo-Businco, Oriano Grossi, Ifigenia Sfika, Giovanni Ragusa, Martina Campisano, Stefano Miceli-Sopo

**Affiliations:** ^1^Allergy Pediatric Unit, Pediatrics Department, Sandro Pertini Hospital, Rome, Italy; ^2^NESMOS Department, Faculty of Medicine and Psychology, Pediatric Unit Sant’Andrea Hospital, “Sapienza” University, Rome, Italy; ^3^Pediatrics Department, Catholic University of Rome, Rome, Italy

**Keywords:** asthma control test, exercise-induced bronchoconstriction, children, parents, questionnaires, lung function, atopy

## Abstract

**Background:**

Poor asthma control can lead to exercise-induced bronchoconstriction (EIB), but the relationship between subjective disease control and EIB is unclear. No studies have compared asthma control test (ACT) scores of children with those of their parents regarding EIB. We assessed whether ACT scores predict the occurrence of EIB in two age groups. We also evaluated ACT scores and objective measures as explanatory variables for airway response to exercise.

**Methods:**

Patients (71, aged <12 years; 93, aged ≥12 years) and their parents completed an ACT questionnaire separately. Current therapy, skin prick testing, and spirometry at baseline and after exercise were assessed. EIB was defined as a fall in forced expiratory volume in 1 s (FEV_1_) of at least 12% from baseline. Sensitivity and specificity for cut-off values of ACT scores predictive of EIB were plotted, and the area under curve (AUC) was described.

**Results:**

Atopy and current therapy were similarly frequent. EIB was observed in 23.9% of children aged <12 years and in 33.3% of children aged ≥12 years. EIB occurrence in subjects previously scored as having full control (25), partial control (20–24), and no control (<20) varied according to the age group and responders. Percentages of EIB cases increased as ACT scores decreased in children aged ≥12 years alone (child ACT scores, 25: 21.9%, 20–24: 31.1%, <20: 62.5%, *p* = 0.017). Plots for ACT scores as predictors of EIB yielded low non-significant AUC values in children aged <12 years; in contrast, moderate AUC values emerged in children aged ≥12 years (child: 0.67, *p* = 0.007; parent: 0.69, *p* = 0.002). Sensitivity of ACT scores below 20 as a predictor of EIB was low in older children (child: 32.3%, parent: 22.6%), whereas specificity was high (child: 90.3%, parent: 93.5%). Multiple regression analysis with percent fall in FEV_1_ as dependent variable included FEV_1_/FVC%, ACT child score, and gender in the prediction model (*r* = 0.42, *p* = 0.000).

**Conclusion:**

ACT scores are a more effective means of excluding than confirming EIB in asthmatic patients aged ≥12 years; their predictive value decreases in younger patients. ACT scores together with lung function may help to predict airway response to exercise. New tools for pediatric asthma assessment may optimize this association.

## Introduction

Exercise-induced bronchoconstriction (EIB) is a transient narrowing of the airways that affects 40–90% of asthmatic children and adolescents (1–3). Prevention of this condition, which is essential in young patients because EIB prevents their participation in vigorous activities (4), can be achieved by means of appropriate asthma therapy (5, 6). As exercise-induced symptoms cannot always diagnose EIB, this pathology is the best documented objectively using a bronchial challenge (3, 7).

Exercise testing is a suitable bronchial challenge for children; exercise-induced hyperpnea indirectly provokes airway narrowing through local dehydration and hyperosmolarity, followed by the release of several inflammatory mediators (8). As with other indirect challenges, EIB is at least partially inhibited by inhaled corticosteroids (ICs) (8, 9); hence, its detection reflects active airway inflammation and helps when therapy needs to be adjusted and the disease monitored (7, 8).

Management of asthma, as stated by international guidelines, is based on the assessment of disease control (10, 11). A useful numerical method to evaluate the level of disease control is the asthma control test (ACT), which includes questions regarding symptoms, medication use, and self-assessed disease control (12). The ACT questionnaire has been validated for subjects over 12 years of age (13). An ACT version for younger children (C-ACT) has also been validated for subjects between 4 and 11 years of age (14).

Questionnaires for assessing asthma control in children provide useful information for research study purposes, though their usefulness in routine clinical practice is still debated (15). One important limitation is the discordance between asthma symptoms reported by the children themselves and those described by the parents (16). However, these contrasting reports may contribute to our understanding of disease control in such patients as estimated on the basis of outcomes from bronchial challenge with exercise.

Few studies have tested the relationship between the occurrence of EIB and the degree of asthma control as assessed by questionnaires that yield contrasting results (17–19), and no studies have compared ACT scores of children with those of their parents regarding EIB. A better knowledge of this issue may shed light on the role played by these scores in the monitoring of asthma either on their own or together with objective measures, such as lung function and exercise testing.

The aim of our study was to assess the ability of ACT scores (yielded by both children and their parents/guardians) to predict the occurrence of EIB in two groups of asthmatic patients divided using an age cut-off of 12 years. We also evaluated ACT scores and objective measures (baseline lung function, atopy, and anthropometric characteristics) as potentially explanatory variables for the airway response to exercise in the whole population.

## Materials and Methods

### Subjects

We assessed 173 asthmatic outpatients aged 7–20 years who came to our pediatric unit (S. P. Hospital) for a follow-up visit from February 2008 to April 2009. Asthma was classified according to global initiative for asthma guidelines (10). Subjects with mild to moderate asthma were invited to participate; they had previously documented bronchial reversibility [a post-bronchodilator forced expiratory volume in 1 s (FEV_1_) increase ≥12%] or a positive response to exercise challenge (fall in FEV_1_ ≥12%). Current asthma therapy was recorded. Subjects who had had any respiratory disorders in the previous 4 weeks were excluded. Informed consent was obtained from the children’s parents. This study was nested within a previous study (20) that was approved by the ethics committee and the ethics committee indicated that no extra approvals were required. In our specialized unit (allergy and pulmonology section), bronchial challenge with exercise and allergen skin prick testing are included as routine tests. The clinical criteria to obtain exercise testing in our institution are either to support a diagnosis of asthma or to assess asthma control; identification of EIB enables us to adjust asthma therapy and, through counseling on preventive measures, to encourage patient participation in motor activities. We routinely assess atopy to classify the asthma phenotype, whose therapeutic implications (e.g., the allergic phenotype respond better to therapy with ICs) are stated in international asthma guidelines (10).

### Study Design

All measurements were performed in a single session. Before exercise testing, current respiratory symptoms and asthma medication were assessed, and patients underwent a medical visit. Parents and children completed an ACT questionnaire on how the patient’s disease was being controlled separately; children also underwent a skin prick test at least 1 h before the exercise challenge. The lung function laboratory personnel were unaware of the questionnaire results. Subjects were divided in two different age groups: below 12 and 12 years or older.

### ACT Questionnaire

The ACT is a validated, five-item, patient-completed measure of asthma control with a 4-week recall period. By summing the five-item scores, three levels of control are identified: scores from 5 to 19 indicate uncontrolled asthma; scores from 20 to 24 indicate partially controlled asthma, and a score of 25 indicates fully controlled asthma (12, 13). To compare self-assessed control and parent-perceived asthma control, both patients aged at least 12 years and their parents were asked to complete the ACT questionnaire blindly. Children aged below 12 years (7.3–11.9 years) completed the ACT questionnaires with the help of the interviewer (whereas their parents completed the ACT blindly) because younger children often need guidance when answering questions (14); the Italian version of the C-ACT was not available at that time.

### Skin Prick Test

The skin prick test was performed using commercial allergens (ALK-Abellò, Milan, Italy) for several common inhaled allergens (*Dermatophagoides pteronissinus* and *D. farinae*, cat and dog fur, *Alternaria alternata*, *Phleum pratense, Cynodon dactylon*, *Plantago lanceolata*, *Chenopodium album*, pellitory, mugwort, ragweed, *Olea europaea*, cypress, birch, plane, elm) and food allergens (milk, egg white, egg yolk, soybean, tomato, codfish, shrimp, wheat, peach, and peanut). Histamine 0.1 mg/ml and glycerol solution were used as positive and negative controls, respectively. Morrow-Brown needles were used to prick the skin, and the wheal reactions were read after 15 min. A wheal ≥3 mm after subtraction of the negative control was regarded as positive (21). The sum of positive skin reactions corrected by the histamine wheal size was termed as prick index (22).

### Spirometry and Exercise Testing

Spirometry was performed with a Pony FX device (Cosmed, Rome, Italy) in the seated position at baseline and after exercise, as recommended (23). Duplicate measurements were obtained from at least three acceptable forced vital capacity (FVC) maneuvers (23) and expressed as a percentage of predicted values (24). Subjects performed an incremental treadmill exercise test as described elsewhere (25), running at 6 km/h with a pendant 10% until they reached a heart rate of between 80 and 90% of the maximum predicted (220 − age in years), according to ATS recommendations for exercise challenge tests in children (26, 27). Room temperature was kept in the 20–24°C range and ambient-relative humidity between 50 and 60%. Spirometry was repeated 5, 10, 15, 20, and 30 min post-exercise. Exercise response was calculated as the maximum post-exercise fall in FEV_1_ expressed as a percentage from baseline. EIB was defined as a fall in FEV_1_ of at least 12% (27).

### Statistical Analysis

Continuous variables were assessed for normal distribution (Kolmogorov–Smirnov test) and expressed consequently as means ± SD or as medians and interquartile (IQR) ranges; categorical variables were given as numbers and percentages. Non-parametric Mann–Whitney tests were used for unpaired comparisons between two groups, and contingence tables (χ^2^ with Fisher’s correction) were used to compare frequencies between categorical variables. Cohen’s κ coefficient was used to estimate the agreement between two ACT raters (child and parent), with κ = 1 indicating perfect agreement and κ ≤ 0 indicating that inter-rater agreement is less than that expected by chance (28). Agreement for intermediate κ values was defined as “poor-to-fair” (<0.40), “moderate” (0.41–0.60), “substantial” (0.61–0.80), and “almost perfect” (0.81–1.0) (27).

The graphical relationship between sensitivity and 1-specificity for all possible cut-off values of ACT scores predictive of EIB was plotted as a receiver operating characteristic (ROC) curve, and the area under curve (AUC) was described. The sample size required for an ROC curve was calculated as described by Hanley and McNeil (29); the number of cases required for an assumed type I error (α: significance) of 0.05, a type II error (β: 1 − power) of 0.2, an expected AUC 0.70, a null hypothesis value 0.5, and a ratio of sample sizes in negative (without EIB)/positive (EIB) groups of 2 was 76. The non-parametric method of DeLong et al. was used to compare the areas under the two ROC curves (30).

Pearson’s or Spearman’s ρ tests were used for correlations as per data distribution type. Stepwise multiple linear regression was performed, with the maximum fall in FEV_1_ as the dependent variable against potential explanatory variables selected on the basis of either statistically significant correlations with the dependent variable or significant differences between categorical variables according to the fall in FEV_1_, as described elsewhere (31). A MedCalc software (MedCalc bvba, Ostend, Belgium) was used for sample size calculation and comparison between the ROC curves; all the remaining statistical analyses were performed using the SPSS software (Version 19; SPSS Inc., Chicago, IL, USA). Two-tailed *p* values of <0.05 were considered statistically significant.

## Results

Nine of the 173 asthmatic subjects who were invited to participate were excluded: 7 were uncooperative during the spirometry or exercise challenge, while 2 refused the skin prick test. The remaining 164 children, who were divided in two age groups (71 aged below 12 years, 93 aged 12 years or above), completed all the measurements. The frequency of atopy and current anti-inflammatory therapy with ICs or oral montelukast was similar in both age groups [patients <12 years, atopy: 63 (88.7%), asthma therapy: 19 (26.8%); patients ≥12 years, atopy: 89 (95.7%), asthma therapy: 21 (22.6%)] (Table 1).

**Table 1:** **Demographics and measurements in the asthmatic patients divided by age group**.

	Age <12 years (*n* = 71)	Age ≥12 years (*n* = 93)
Males, *n* (%)	48 (67.6)	63 (67.7)
Age, years	10.0 ± 1.2	14.3 ± 1.8**
Height, cm	142.0 ± 8.2	163.0 ± 10.1**
Weight, kg	39.2 ± 9.6	60.5 ± 11.8**
Atopy, *n* (%)	63 (88.7)	89 (95.7)
Prick index, inhalants^a^	4.1 ± 2.8	4.3 ± 2.5
Prick index, foods^b^	0.7 ± 1.2	0.8 ± 1.1
Passive smoke, *n* (%)	26 (36.6)	25 (26.9)
Therapy, *n* (%)^c^	19 (26.8)	21 (22.6)
ICs	17 (23.9)	18 (19.4)
Montelukast	6 (8.5)	10 (10.8)
FEV_1_, % predicted	99.4 ± 13.1	102.5 ± 13.1
FVC, % predicted	105.1 ± 11.7	107.9 ± 13.6
FEV_1_/FVC, %	85.4 ± 7.5	83.6 ± 6.7
PEF% predicted	104.6 ± 16.4	108.9 ± 19.4
FEF_25–75_, % predicted	79.9 ± 24.4	83.7 ± 22.6
ACT child	22.0 (20.0–24.0)	23.0 (21.0–25.0)*
ACT parent	23.0 (20.0–25.0)	24.0 (22.0–25.0)

*Frequencies are expressed as number and percentage; continuous variables are expressed as arithmetic mean ± SD or as median (interquartile range)*.

*p < 0.05 and **p < 0.01 vs. subjects aged <12 years.

*Prick index^a,b^: sum of allergen skin-wheal reactions for common inhalants or foods, corrected by the histamine wheal size (millimeters). ^c^Current therapy with inhaled corticosteroids (ICs) and/or montelukast*.

*EIB was defined as a post-exercise fall in FEV_1_ ≥12% from baseline*.

*ACT, asthma control test score*.

### Agreement between Child and Parent ACT Scores

Scores yielded by the ACT completed by children differed from those of the ACT completed by their parents, particularly in the group aged below 12 years. The percentages of concordant child vs. parent responses for all the ACT score intervals (uncontrolled: <20, partially controlled: 20–24, fully controlled: 25) were 56.3% in the younger group [κ (SE) agreement 0.295 (0.097), *p* = 0.000] and 75.3% in the older group [κ (SE) agreement 0.598 (0.073), *p* = 0.000]. According to the κ values, agreement between the younger age group and their parents was “poor-to-fair,” whereas agreement between the older age group and their parents was “moderate” (Table 2).

**Table 2:** **Agreement between child and parent ACT scores according to age group**.

		ACT parent					
		<12 years (*n* = 71)			≥12 years (*n* = 93)		
		<20	20–24	25	<20	20–24	25
ACT child	<20	**8**	9	0	**10**	5	1
	20–24	3	**21**	14	1	**32**	12
	25	0	5	**11**	0	4	**28**

*Numbers of concordant child- vs. -parent responses for intervals of ACT scores are given in bold type*.

*<12 years: 8 + 21 + 11/71 (56.3%), κ (SE) agreement 0.295 (0.097), *p* = 0.000*.

*≥12 years: 10 + 32 + 28/93 (75.3%), κ (SE) agreement 0.598 (0.073), *p* = 0.000*.

### Occurrence of EIB

A post-exercise fall in FEV_1_ of at least 12% (EIB) was observed in 17 (23.9%) of the children aged below 12 years and in 31 (33.3%) of the children aged 12 years or above. Subjects with EIB were more frequently treated with asthma medication and had lower baseline lung function and ACT scores than children without EIB, though differences for ACT scores were significant in the older group alone (Table 3).

**Table 3:** **Characteristics, measurements, and ACT scores according to presence of exercise-induced bronchoconstriction (EIB) in the asthmatic patients divided by age group**.

	Age <12 years		Age ≥12 years	
	Without EIB (***n*** = 54)	EIB (*n* = 17)	Without EIB (*n* = 62)	EIB (*n* = 31)
Males, *n* (%)	39 (72.2)	9 (52.9)	43 (69.4)	20 (64.5)
Age, years	10.0 ± 1.2	9.7 ± 1.2	14.4 ± 1.8	14.1 ± 2.0
Height, cm	141.9 ± 8.4	142.1 ± 7.9	163.9 ± 10.3	161.1 ± 9.3
Weight, kg	39.8 ± 10.1	37.4 ± 8.0	60.7 ± 11.8	60.1 ± 12.1
Atopy, *n* (%)	47 (87.0)	16 (94.1)	58 (93.5)	31 (100.0)
Prick index, inhalants^a^	4.3 ± 2.8	3.7 ± 2.5	4.7 ± 2.7	3.7 ± 2.2
Prick index, foods^b^	0.8 ± 1.3	0.4 ± 0.7	0.8 ± 1.2	0.7 ± 1.0
Passive smoke, *n* (%)	20 (37.0)	6 (35.3)	18 (29.0)	7 (22.6)
Therapy, *n* (%)^c^	12 (22.2)	7 (41.2)	7 (11.3)	14 (45.2)**
ICs	10 (18.5)	7 (41.2)	6 (9.7)	12 (38.7)**
Montelukast	5 (9.3)	1 (5.9)	3 (4.8)	7 (22.6)*
FEV_1_, % predicted	101.6 ± 12.3	92.6 ± 13.6*	104.3 ± 13.8	98.9 ± 11.1*
FVC, % predicted	105.8 ± 12.2	102.9 ± 9.9	108.3 ± 13.9	106.9 ± 13.3
FEV_1_/FVC, %	86.5 ± 6.8	81.8 ± 8.7*	84.4 ± 6.4	81.9 ± 7.0
PEF% predicted	106.3 ± 17.1	99.5 ± 13.1	111.0 ± 19.0	104.8 ± 19.8
FEF_25–75_, % predicted	82.9 ± 24.4	70.6 ± 22.7	87.0 ± 23.2	76.9 ± 20.1*
ACT child	22.0 (20.0–24.0)	22.0 (19.0–25.0)	24.0 (22.0–25.0)	22.0 (18.0–24.0)**
ACT parent	23.0 (20.0–25.0)	22.0 (20.0–24.0)	24.5 (23.0–25.0)	21.0 (20.0–25.0)**

*Frequencies are expressed as number and percentage; continuous variables are expressed as arithmetic mean ± SD or as median (interquartile range)*.

*p < 0.05 and **p < 0.01 vs. subjects without EIB from the same group.

*Prick index^a,b^: sum of allergen skin-wheal reactions for common inhalants or foods, corrected by the histamine wheal size (millimeters). ^c^Current therapy with inhaled corticosteroids (ICs) and/or montelukast*.

*EIB was defined as a post-exercise fall in FEV_1_ ≥12% from baseline*.

*ACT, asthma control test score*.

### Distribution of EIB According to Levels of Asthma Control

**Figure 1:**
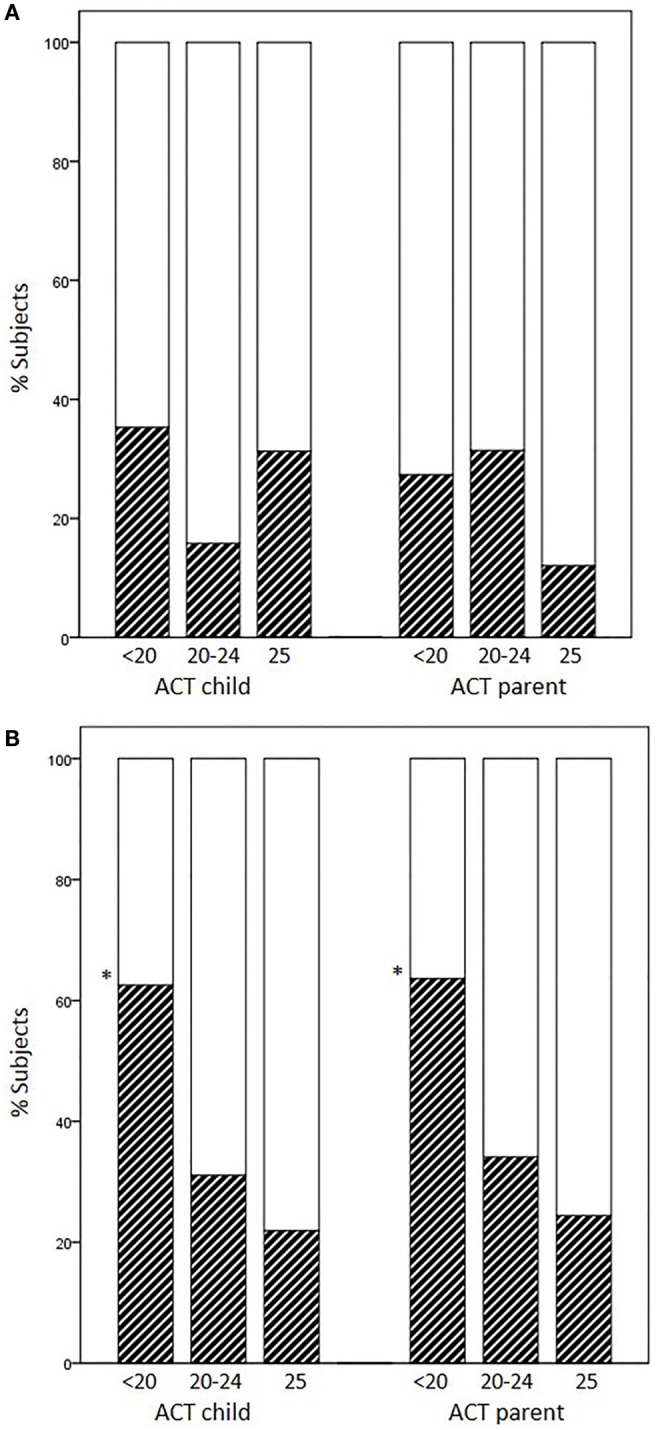
**Percentages of EIB cases according to disease-control level: fully controlled (25), partially controlled (20–24), and uncontrolled (<20)**. **(A)** Patients aged <12 years, **(B)** patients aged ≥12 years (*p = 0.017 and *p* = 0.049 for child and parent ACT, respectively).

Occurrence of EIB in subjects previously scored as having full control (25), partial control (20–24), and no control (<20) varied according to the age group and responder (child or parent). The percentages of EIB cases divided according to each disease-control level (positive/negative + positive × 100) did not increase as ACT scores decreased in younger children, whereas they did increase in children aged 12 years or above (child ACT scores, 25: 21.9%, 20–24: 31.1%, <20: 62.5%, *p* = 0.017; parent ACT scores, 25: 24.4%, 20–24: 34.1%, <20: 63.6%, *p* = 0.049) (Figures 1A,B).

### ACT Scores as Predictors of Occurrence of EIB According to Age Group

**Figure 2:**
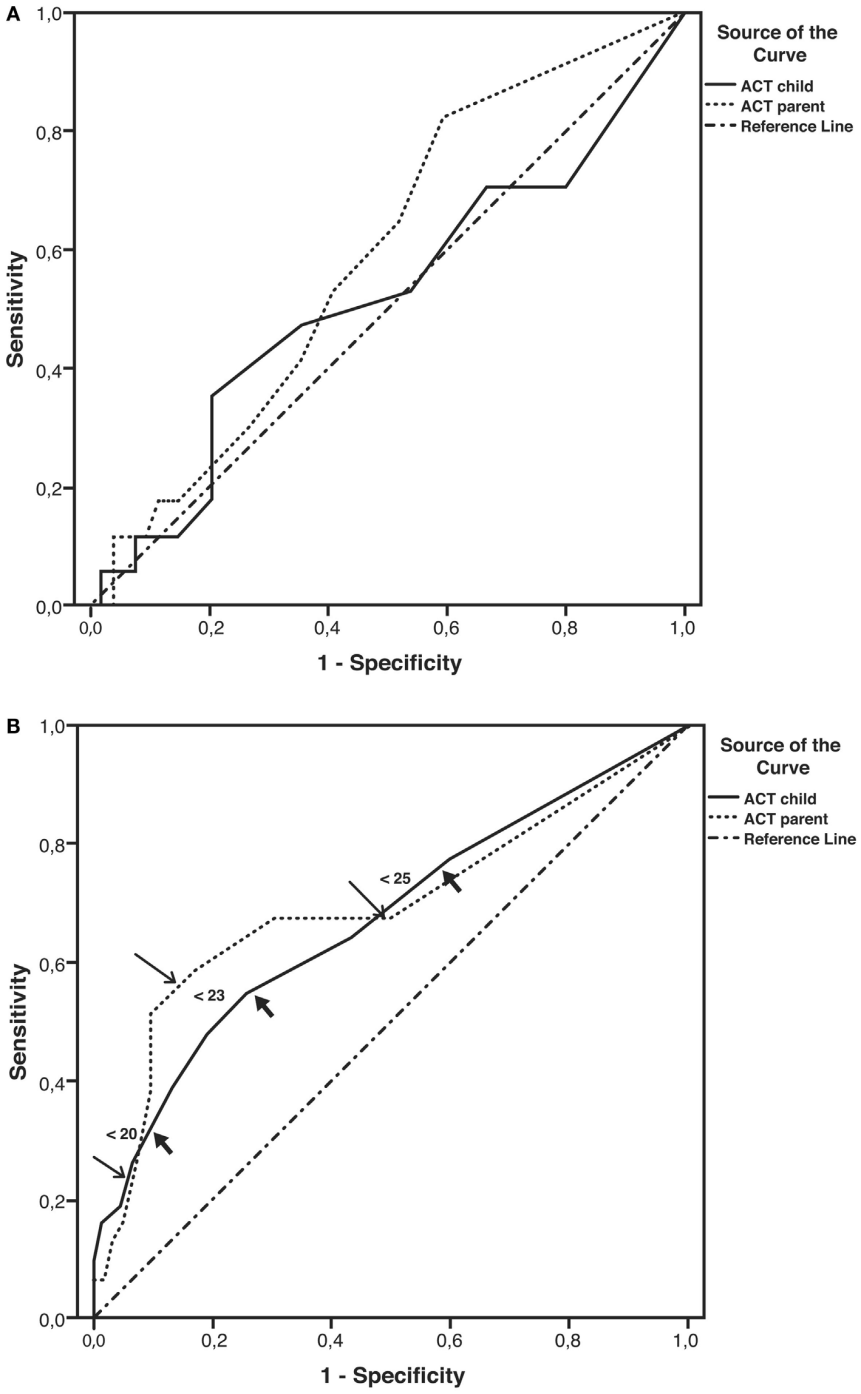
**Receiver operating characteristic (ROC) curves for ACT scores as predictors of EIB**. **(A)** Patients aged under 12 years; areas under curves (AUCs): child: 0.52, *p* = 0.814; parent: 0.59, *p* = 0.255. **(B)** Patients aged ≥12 years; AUCs: child: 0.67, *p* = 0.007; parent: 0.69, *p* = 0.002; thick arrows (child) and thin arrows (parent) indicate predictive values for ACT scores below 20, 23, and 25 (detailed in Table 4).

Sensitivity and specificity plots for cut-off points of the ACT scores as predictors of EIB (ROC curves) yielded low AUC values in the group aged below 12 years (child: 0.52, *p* = 0.814; parent: 0.59, *p* = 0.255); in contrast, moderate AUC values emerged in the group aged 12 years or above (child: 0.672, *p* = 0.007; parent: 0.695, *p* = 0.002). The sensitivity of ACT scores below 20 (loss of control) as a predictor of EIB was low in the older age group, particularly in parents (child: 32.3%, parent: 22.6%), whereas specificity was high (child: 90.3%, parent: 93.5%). The sensitivity of ACT scores below the intermediate values within the 20–24 range (partial control) as predictors of EIB improved (ACT <23, child: 54.8%, parent: 58.1%), whereas the specificity declined, particularly in children (ACT <23, child: 74.2%, parent: 83.9%) (Table 4). However, no significant differences were detected between older children and their parents in the ROC curves (difference between areas 0.0234, 95% CI –0.0732 to 0.120, *p* = 0.6349) (Figures 2A,B; Table 4).

**Table 4:** **Prediction of EIB in subjects with loss of asthma control (ACT score <20) and subjects scored below full disease control (<23, <25)**.

	Age <12 years (*n* = 71)			Age ≥12 years (*n* = 93)		

ACT score child	<20	<23	<25	<20	<23	<25
Sensibility	35.3	52.9	70.6	32.3	54.8	77.4
Specificity	79.6	46.3	20.4	90.3	74.2	40.3
PPV	35.3	23.7	21.8	62.5	51.5	39.3
NPV	79.6	75.8	68.7	72.7	76.7	78.1

**ACT score parent**	**<20**	**<23**	**<25**	**<20**	**<23**	**<25**

Sensibility	17.6	52.9	82.3	22.6	58.1	67.7
Specificity	85.2	59.3	40.7	93.5	83.9	50.0
PPV	27.3	29.0	30.4	63.6	64.3	40.4
NPV	76.7	80.0	88.0	70.7	80.0	75.6

*EIB, exercise-induced bronchoconstriction (post-exercise fall in FEV_1_ ≥12% from baseline); ACT, asthma control test; PPV, positive predictive value; NPV, negative predictive value*.

### Assessment of Influencing Factors on Airway Response to Exercise in the Whole Population

The percent fall in FEV_1_ following exercise correlated with low baseline lung function, and ACT scores but did not correlate with atopy scores for inhalant or food allergens (Table 5). A multiple regression analysis with the percent fall in FEV_1_ as the dependent variable against potential explanatory variables included FEV_1_/FVC%, ACT child score, and gender (male = 0, female = 1) in the prediction model (*r* = 0.42, *p* = 0.000):

% fall in FEV1=−85.208+(0.705×FEV1/FVC%)+(ACT child×0.739)+(−4.880×gender).

**Table 5:** **Spearman’s ρ correlations with the post-exercise fall in FEV_1_ in the whole population (*n* = 164 asthmatic children)**.

Variable	Correlation (*r*)	*p* Value
Age, years	–0.005	0.945
Height, cm	–0.009	0.913
Weight, kg	–0.020	0.803
Prick index, inhalants^a^	0.138	0.102
Prick index, foods^b^	–0.031	0.709
FEV_1_, %	0.166	0.033
FVC, %	0.017	0.824
FEV_1_/FVC, %	0.169	0.030
PEF%	0.158	0.043
FEF_25–75_, %	0.162	0.038
ACT child	0.141	0.072
ACT parent	0.186	0.017

*ACT, asthma control test score*.

*Prick index^a,b^: sum of allergen skin-wheal reactions for common inhalants or foods, corrected by the histamine wheal size (millimeters)*.

## Discussion

We found that ACT scores, as completed by children or their parents, were moderately good predictors of EIB in our group of asthmatic patients aged 12 years and older but poor predictors of EIB in patients under 12 years of age. Subjective information from the ACT was used to complement data obtained from objective measures, such as baseline lung function and gender, to explain the airway response to exercise in the whole population.

We analyzed ACT scores from patients above and below the 12-year-old cut-off separately to ensure that questionnaires were applied to the recommended age range, at least for the older age group. Since an Italian version of the C-ACT for children under 12 years of age was not available when we conducted our study, we asked both children and their parents to complete the questionnaires in both age groups. Not only did we expect the parent’s perception of their children’s asthma control to compensate for the inadequacies of the ACT but we were also interested in examining the inter-rater agreement (child vs. parent) of the questionnaire scores. Since agreement for the ACT score intervals between young patients and their parents was, according to the κ values, “poor-to-fair,” our results indicate that asking parents to complete the ACT questionnaires cannot be considered as a reliable surrogate of the C-ACT in asthmatic children younger than 12 years of age.

The relationship between ACT scores and EIB has rarely been assessed in asthmatic children, particularly in those aged 12 years and above. No studies have compared the value of patients’ and parents’ responses to the ACT as a predictor of EIB. The relationship between the ACT and EIB has previously been reported for a group that included pediatric patients whose age ranged widely (17). Rapino et al. assessed self-completed ACT questionnaires in 81 asthmatic children aged 6–17 years who performed an exercise challenge; EIB (defined as a fall in FEV_1_ >10%) was no more frequent in subjects whose score indicated uncontrolled asthma (ACT <20); moreover, subjects with fully controlled asthma (ACT =25) more frequently had EIB than subjects with partially controlled and uncontrolled asthma together (36.0 vs. 23.5%, *p* < 0.01) (17). In contrast to their study, we defined EIB as a fall in FEV_1_ >12% and analyzed our population according to age groups rather than as a whole. Our results are in keeping with those of Rapino et al. solely for our group of patients aged below 12 years. In contrast, the likelihood of EIB increased as ACT scores decreased in our patients aged 12 years or above regardless of whether it was the children or their parents who completed the ACT. Our results pointing to the low sensitivity and high specificity of ACT scores as predictors of EIB in cases of uncontrolled asthma in our older age group suggest that the ACT is more effective as a means of excluding, rather than confirming, EIB.

Possible correlations between the EIB and other questionnaires, such as the C-ACT and asthma control questionnaire (ACQ), have also been assessed (18, 19). Chinellato et al. found a moderately good discriminatory power of the C-ACT total score as a predictor of EIB in young children aged 4–11 years, particularly as a predictor of the absence of EIB in subjects rated above 19, i.e., with partial-to-full disease control (18). Our study did not include very young children (4–6 years), whose recall difficulty beyond 1 day is well known (14). The frequency of EIB in our young patients who rated themselves 20 or higher (11/54 = 20.4%) was similar to that reported by Chinellato et al. (14/72 = 19.4%). Moreover, an ACT score <20 in our young patients was a moderately good predictor of EIB (sensitivity 35%, specificity 80%), whereas a score from 20 to 24 (e.g., <23) was a poor predictor of EIB. Consequently, no significant areas were detected when the ROC curves for EIB and for the ACT scores yielded by our young patients and their parents were compared.

We used the ACT questionnaire without adding questions on exercise-induced symptoms. Unlike the ACT, some questionnaires in young children (e.g., TRACK and C-ACT) inquire about activity limitation (18, 32). However, Chinellato et al. did not detect any relationship between scores for the single C-ACT question on exercise-related problems and the degree of EIB in their young subjects, while Rapino et al. found that a direct question on exercise-induced symptoms (in addition to the ACT questionnaire) did not help to discriminate subjects with EIB (17). These reports further support the notion that self-reported exercise-induced symptoms are not very reliable as a means of predicting EIB (7, 27).

In contrast to studies based on the ACT and C-ACT, Madhuban et al. found no relationship between the categorical ACQ and the occurrence of EIB in 200 asthmatic children; the authors pointed out that 41% of their children with well-controlled asthma, according to the ACQ, had EIB, thus implying that their asthma was not well controlled (19). Although previous results are not encouraging, the potential usefulness of questionnaires as a means of ruling out airway hyperresponsiveness to exercise cannot be excluded.

An interesting question raised by our results is why ACT scores obtained from parents differ from those of children, and which are more reliable. One-third of the children who responded as having “no asthma control” (<20) had EIB, whereas a quarter of the parents’ scores <20 predicted that their child had EIB. When the ACT cut-off values were raised to <23 (which includes low scores of “partial” asthma control plus “no asthma control”), the parents’ scores slightly improved prediction of EIB if compared with those of their children. This discordance suggests that some parents play down the effectiveness of disease control in their children. As AUCs did not differ between children and their parents, we are unable to recommend the use of parent-completed ACT responses for the prediction of EIB in their children aged 12 years or above.

Asthma control test scores obtained from children responses together with the baseline FEV_1_/FVC% and gender explained the change in FEV_1_ following exercise in our overall population. Reports on the relationship between baseline lung function and gender in cases of post-exercise airway narrowing are contrasting (2, 7, 33–36). Baseline FEV_1_ did not explain the degree of EIB in two studies (2, 33), whereas the baseline FEV_1_/FVC did in another report, which is in keeping with our results (34). The prevalence of EIB was slightly higher in females than in males in some studies that assessed unselected populations (7, 36). Males accounted for about two-thirds of our asthmatic population and for 60% of the EIB cases, a fact that might have biased our results. Another bias could be caused by the fact that subjects who performed exercise testing successfully may not represent the entire asthmatic population; indeed, our selection criteria implied that subjects unable to cooperate (e.g., young age, refusal/inadequate performance of testing procedures) were excluded. Nonetheless, our data suggest that questionnaire-based assessments may be used to complement objective measurements to predict asthma control according to an exercise bronchial challenge.

In conclusion, ACT scores are a more effective means of excluding than of confirming EIB if used in asthmatic patients aged 12 years and older; their predictive value decreases in younger patients, even when the ACT questionnaire is completed by their parents. Subjective information gleaned from the ACT together with objective measures, such as lung function and gender, may help to predict the airway response to exercise, and consequently, to estimate disease control and adjust therapy, accordingly. Further pediatric studies, preferably designed according to new pediatric asthma assessment tools, are warranted to optimize subjective measures of asthma control and to assess their relationship with EIB.

## Author Contributions

ST setup and managed the study and recruited the participants. ST and MB interpreted the data. MB revised and analyzed the data and wrote the final version of the manuscript. AR-B, OG, and GR recruited participants and performed exercise tests. MC collected data and helped to critically revise the work. SM-S conceptualized the study. IS and GR managed and cleaned the database. ST, AR-B, SM-S, OG, and IS co-drafted the initial version of the manuscript. All authors read and approved the present version of the manuscript.

## Conflict of Interest Statement

The authors declare that the research was conducted in the absence of any commercial or financial relationships that could be construed as a potential conflict of interest.

## Abbreviations

ACT, asthma control test; AUC, area under curve; EIB, exercise-induced bronchoconstriction; FEV_1_, forced expiratory volume in 1 s; FVC, forced vital capacity; ROC, receiver operating characteristic curve.
